# Neoadjuvant chemotherapy modulates exhaustion of T cells in breast cancer patients

**DOI:** 10.1371/journal.pone.0280851

**Published:** 2023-02-10

**Authors:** Ivon Johanna Rodríguez, David A. Bernal-Estévez, Manuela Llano-León, Carlos Eduardo Bonilla, Carlos Alberto Parra-López

**Affiliations:** 1 Departamento de Microbiología, Laboratorio de Inmunología y Medicina Traslacional, Facultad de Medicina, Universidad Nacional de Colombia, Bogotá, Colombia; 2 Departamento de Movimiento Corporal Humano, Facultad de Medicina, Universidad Nacional de Colombia, Bogotá, Colombia; 3 Immunology and Clinical Oncology Research Group (GIIOC), Fundación Salud de los Andes, Bogotá, Colombia; 4 Departamento de Química, Facultad de Ciencias, Universidad Nacional de Colombia, Bogotá, Colombia; 5 Departamento de Oncología Clínica, Instituto Nacional de Cancerología, Bogotá, Colombia; Kansai Medical University: Kansai Ika Daigaku, Institute of Biomedical Science, JAPAN

## Abstract

Breast cancer is the leading cause of cancer deaths in women worldwide. It has been observed that the incidence of breast cancer increases linearly with age after 45, which suggest a link between cancer, aging, and senescence. A growing body of evidence indicates that the immunosuppressive tumor network in breast cancer patients can lead to T-cell exhaustion and senescence. Cytotoxic chemotherapy is a common treatment for many cancers, and it is hypothesized that its efficacy may be related to immune activation. However, the effects of neoadjuvant chemotherapy on T-cell dysfunction in breast cancer patients are not fully understood. This study aimed to evaluate the impact of neoadjuvant chemotherapy on the expression of exhaustion and senescence markers in T cells in women with breast cancer. Our results showed that T cells from breast cancer patients have a reduced ability to respond to stimulation in-vitro and an increased expression of senescence and exhaustion-associated markers, such as TIM-3, LAG3, and CD57. Furthermore, we found that neoadjuvant chemotherapy has an immunomodulatory effect and reduces the expression of exhaustion markers. Our observations of the immune phenotype of T cells during neoadjuvant chemotherapy treatment highlight its ability to stimulate the immune system against cancer. Therefore, monitoring the response of T cells during chemotherapy may enable early prediction of clinical response.

## Introduction

Breast cancer (BC) is a complex and heterogeneous disease [1]. In 2018, approximately 2.1 million cases of BC were diagnosed, accounting for nearly one in every four cancer cases among women [2]. After 45 yr, the incidence increases linearly with age, with the highest rates after 60 yr [3]. Studies suggest a link between cancer and aging. It is likely that genetic (cancer mutations) and epigenetic (senescent cell accumulation) events contribute to the exponential increase in cancer that occurs with aging [4]. Senescent cells that accumulate with age provide the ideal environment for tumor cells to proliferate [5, 6].

There is evidence to support the role of the immune system (IS) in the prevention and control of tumor progression (immunosurveillance) [7]. However, the IS can also promote tumor development via an immune escape mechanism [8]. The two faces of the IS in tumor development have given rise to the modern concept of cancer immunoediting, which includes three phases (the three Es): elimination, equilibrium, and escape [8–10]. Immunological defects associated with aging may have a direct impact on immunoediting capacity [5]. Immunoaging is associated with less senescent cell clearance, favoring chronic diseases and increasing cancer susceptibility [11]. T cells become less efficient and unable to proliferate as the IS ages and enter replicative senescence, which is associated to telomere shortening and telomerase inactivation [6, 12, 13].

The tumor microenvironment (TME) can also impact T cell function by inducing phenotypes of anergy, exhaustion, or senescence [14]. Anergic T cells have low co-stimulatory molecule expression and high co-inhibitory signaling, do not respond to activation, and produce limited amounts of IL-2 and other interleukins [14]. Exhausted T cells are effector lymphocytes that express several inhibitory receptors (PD1, LAG3, CTLA4, and TIM3) and are therefore unable to proliferate and produce cytokines [15]. Senescent T cells are terminally differentiated cells with decreased CD28 expression and increased CD57 and KLRG1 expression that can produce large amounts of proinflammatory cytokines, similar to the senescence-associated secretory phenotype [14, 16]. The exhaustion and senescence markers can co-express in T cells, implying that a lymphocyte can be exhausted and senescent [15], and they can be present and coexist in the TME, promoting tumor cell progression [17].

Despite advances in immuno-oncology, chemotherapy remains the primary treatment option for different types of cancer at various stages of the disease [18]. The most commonly used treatment for BC is neoadjuvant chemotherapy (NAC) [19]. Chemotherapy effectiveness is based not only on cytotoxic effects on tumor cells but also on the activation of the IS [18], increasing DC maturation, decreasing the Treg and MDSC population, and restoring the cytotoxic activity of T and NK cells [20, 21]. In a recent study, Bernal et al. designed an in-vitro system for monitoring in PBMCs from BC patients, and found that neoadjuvant therapy restores communication between T cells and APCs and correlates with tumor immune surveillance recovery [22, 23]. Different peripheral blood studies may reflect immune-related changes found in the TME [24].

In this work, we evaluated T cells in BC patients before and after chemotherapy to identify the effect of neoadjuvant chemotherapy on T cell immunosuppressive phenotypes associated with senescence and exhaustion. We found that T cells from BC patients have lower internalization capacity of central molecules of the TCR signaling machinery, namely CD3 and pZAP70, and demonstrate higher expression of exhaustion markers, such as TIM3 and LAG3. Neoadjuvant chemotherapy could restore T cells to levels seen in healthy women.

## Materials and methods

### Patients and blood samples

The study was approved by the ethics committee of the Instituto Nacional de Cancerología–Bogotá (reference number ACT-043 No 2018). We invited 31 patients with a pathological breast cancer diagnosis to participate in the study. Seventeen agreed to donate blood samples; of these patients, 13 completed the blood samples before and after chemotherapy and were included in the study (Table 1). All patients were treated with Doxorubicin and Cyclophosphamide (A/C) as neoadjuvant chemotherapy, and ten age-matched healthy women were included. All patients and HDs gave written informed consent before study entry. Afterward, peripheral venous blood (20 mL) was obtained in heparinized tubes. The patients donated two blood samples (first before starting chemotherapy and the second after the third dose of A/C chemotherapy). PBMCs were isolated using a density gradient (Histopaque, Sigma Aldrich) and were cryopreserved in AIM-V 50% (Gibco, ThermoFisher), FBS 40% (Gibco, ThermoFisher), and DMSO 10% (MP Biomedicals, LLC) in liquid nitrogen until use.

**Table 1:** Patients’ clinical characteristics.

Patient number	Age	HER2 (+/-)	PR (%)	ER (%)	Ki67 (%)	Tumoral stage	RCB	RCB Class
1	43	Negative	70	80	40	IB	1,495	RCB-II
2	63	Positive	90	100	15	IB	1,568	RCB-II
3	56	Positive	60	60	15	IIB	0	pCR
4	66	Positive	0	0	40	IIIA	1,342	RCB-I
5	63	Negative	10	100	20	IIIB	4,14	RCB-III
6	42	Negative	100	100	10	IB	1	RCB-II
7	84	Negative	0	0	70	IIIB	4	RCB-III
8	72	Negative	90	100	30	IIIB	1,965	RCB-II
9	62	Negative	2	100	10	IIIB	3.695	RCB-III
10	58	Negative	80	100	88	IIIB	3,380	RCB-III
11	55	Negative	100	100	80	IIA	1,427	RCB-II
12	59	Negative	100	80	60	IIIB	3,685	RCB-III
13	47	Negative	40	40	97	IIA	1,320	RCB-I

HER2 (±), overexpression of HER-2; PR (%), progesterone receptor expression in percent; ER (%), estrogen receptor expression in percentage; Ki67 (%), fraction of cancer cells positive for Ki67 expression; (RCB) Residual Cancer Burden and (RCB class) Residual Cancer Burden class.

### Flow cytometry

The PBMC of each study participant (patients and HD) were thawed and washed in AIM-V medium (Gibco, ThermoFisher) to be used in all experiments. One million PBMCs were seeded in 96-well flat-bottom dishes for 72 hours with a mixture of beads coupled to antibodies against CD3, CD28, and CD2 (Miltenyi Biotec) in a 2:1 ratio (PBMCs: beads) grown in AIM-V medium (Thermo Fisher Scientific). After incubation, the cells were washed with PBS and labeled with one of the following monoclonal antibody panels from Biolegend: (i) *Expression of exhaustion and senescence markers in T cells and internalization of CD3*. Pacific blue TM anti-CD3, Brillant violet 510TM anti-CD4 (SK3), PE / Dazzle594 anti-CD8 (SK1), FITC anti-CD45RO (UCHL1), PerCP / Cy5.5 anti-PD1 (EH12.2H7), PE / Cy7 anti-CTLA4 (L3D10), PE anti-TIM3 (F38-2E2), and APC / FireTM750 anti-KLRG1 (SA231A2). BD bioscience: BV711 anti-CD62L (DREG-56), APC anti-CD57 (NK-1), and BV786 anti-LAG3 (T47-530). (ii) *ZAP70 phosphorylation and Ki-67 expression in T cells*. Pacific blue TM anti-CD3, PE / Dazzle594 anti-CD8 (SK1), APC anti-CD45RA and APC/Cy7 anti-CD27. Intracellular labeling was performed following the instructions of the DAKO Intrastain Kit for PE anti-Ki-67 (11F6) and Alexafluor647 anti-pZAP70 (1503310). It was incubated for 20 min at 4°C. All anti-human antibodies were used at the concentrations recommended by the manufacturer. Flow cytometry data were acquired using FACS Aria IIIu (BD) and analyzed using FlowJo Software V10 (BD).

### Statistical analysis

Statistical analyzes were performed on Prism V9 software (GraphPad). To compare groups (BC vs. HD), we applied non-parametric tests, Mann-Whitney and Kruskal-Wallis tests. In addition, we used the Wilcoxon test for paired samples (Before vs. After) to test for statistical differences p values < 0.05 were considered statistically significant.

## Results

### Chemotherapy restores T cell responsiveness in patients with BC

T cell cellular exhaustion caused by antigenic stimulation is accompanied by TCR activation dysfunction. However, it has been reported that BC patients with T cell dysfunction recover after NAC antitumor therapy, suggesting that chemotherapy has an immunomodulatory role [22].

**Fig 1:**
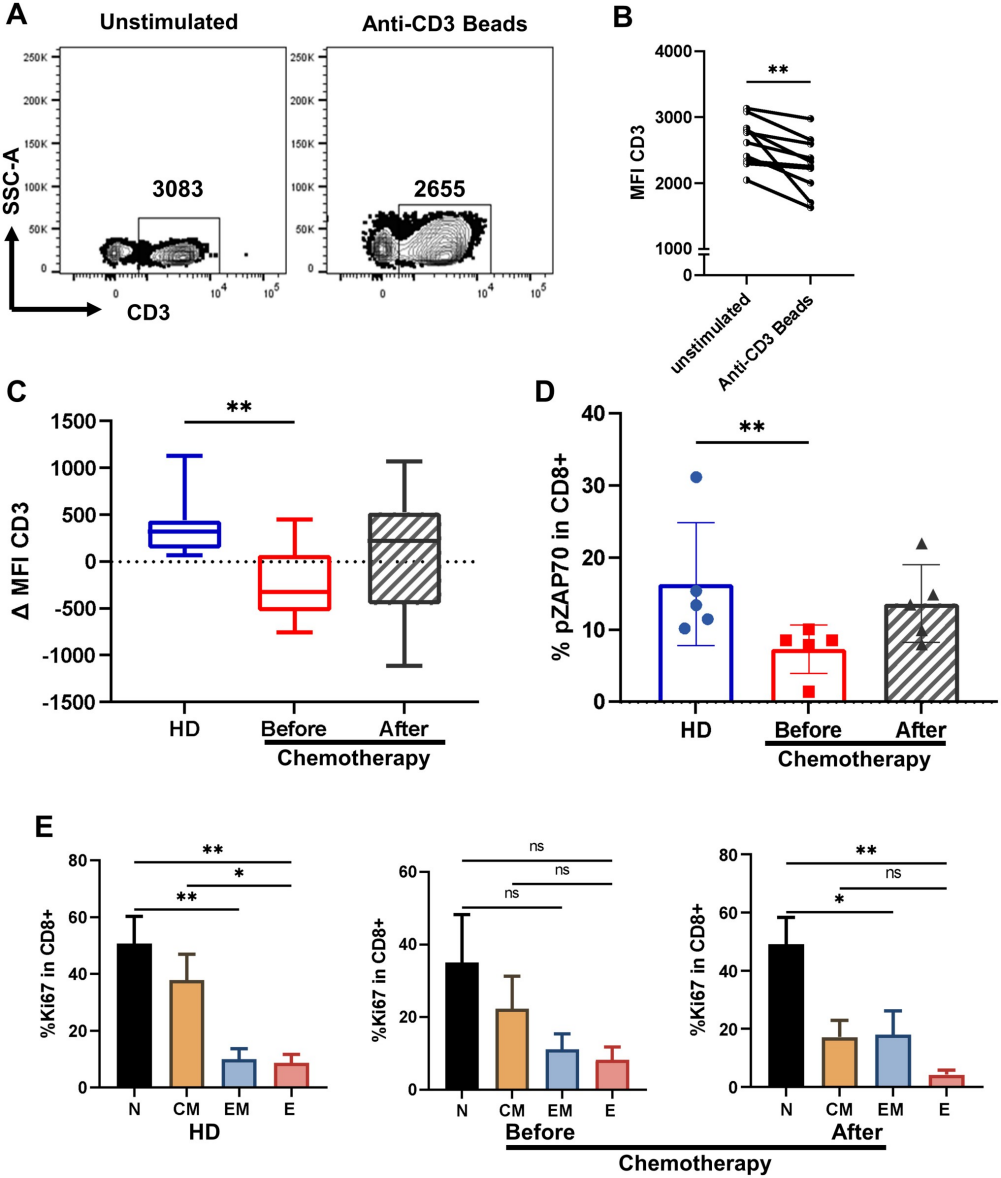
Chemotherapy restores deficient response of T cells in breast cancer patients. **A**. Contour plots from a representative donor’s PBMCs showing SSC-A vs CD3 in lymphocytes (by SSC-A vs FSC-A) after 72 h of unstimulated (left) vs after polyclonal stimulation with anti-CD3/CD28/CD2 micro beads (right), numbers in gates represents the mean fluorescence intensity (MFI) of CD3 measured by flow cytometry. **B**. Paired analysis of CD3 MFI quantification in 10 HD PBMCs unstimulated and after *in-vitro* stimulation for 72 hours with anti-CD3/CD28/CD2 micro beads. **C**. Quantification of percentage of CD3 internalization in HD (blue box), BC patients before (red box) and after chemotherapy (gray box with stripe). The dispersion of the data in boxes and whiskers (5–95%) is shown. **D**. pZAP70 expression in CD8+ T cells after stimulation (n = 5/group). **E**. Ki67 expression in CD27+CD45RA+ naïve T cells (**N** cells), CD27+CD45RA− central memory T cells (CM cells), CD27−CD45RA− effector memory T cells (EM cells), and CD27−CD45RA+ effector T cells (E cells) isolated from PBMCs (n = 10/group). A nonparametric *t-test* was performed with unpaired Mann-Whitney test data to compare HD and BC patient’s. A paired nonparametric Wilcoxon test to compare BC patients before and after chemotherapy. Data presented as means ± SEM (** p <0.01; * p <0.05).

In line with these findings, we conducted an in-vitro assay to assess CD3 internalization as a measure of TCR activation. PBMCs from patients and healthy controls were stimulated for 72 h with anti-CD3/CD28/CD2 antibody-coupled beads. The percentage of CD3 internalization is proportional to the difference in MFI between control and stimulated samples (Fig 1). T cells from HD showed efficient TCR internalization, as evidenced by a decrease in MFI of CD3 after stimulation (Fig 1A and 1B).

In contrast, patients before chemotherapy have lower CD3 internalization capacity, which tends to recover to the level of healthy patients after chemotherapy (Fig 1C). Additionally, we examined the phosphorylation of ZAP70, an important molecule in TCR signaling, and discovered that BC patients have lower ZAP70 phosphorylation than healthy controls (Fig 1D). Subsequently, we measured Ki67 expression to compare the proliferative capacity of whole CD8+ and CD4+ T cells in HD and BC patients before and after NAC. We found no differences between groups (S1A Fig). In contrast, when we evaluate the memory subsets, we found that terminal effector T cells from HD and BC patients after chemotherapy had significantly decreased proliferation compared with less differentiated subpopulations. These results contrast with the degree of proliferation observed in T cells from BC patients before chemotherapy, which revealed no statistically significant differences in proliferation capacity between memory subpopulations (Fig 1E and S1B Fig). Moreover, when we measured cytokine production, we found no significant difference between groups in CD8 T cells (S2 Fig).

### Chemotherapy-induced changes in CD4+ and CD8+ T cell memory subsets

**Fig 2:**
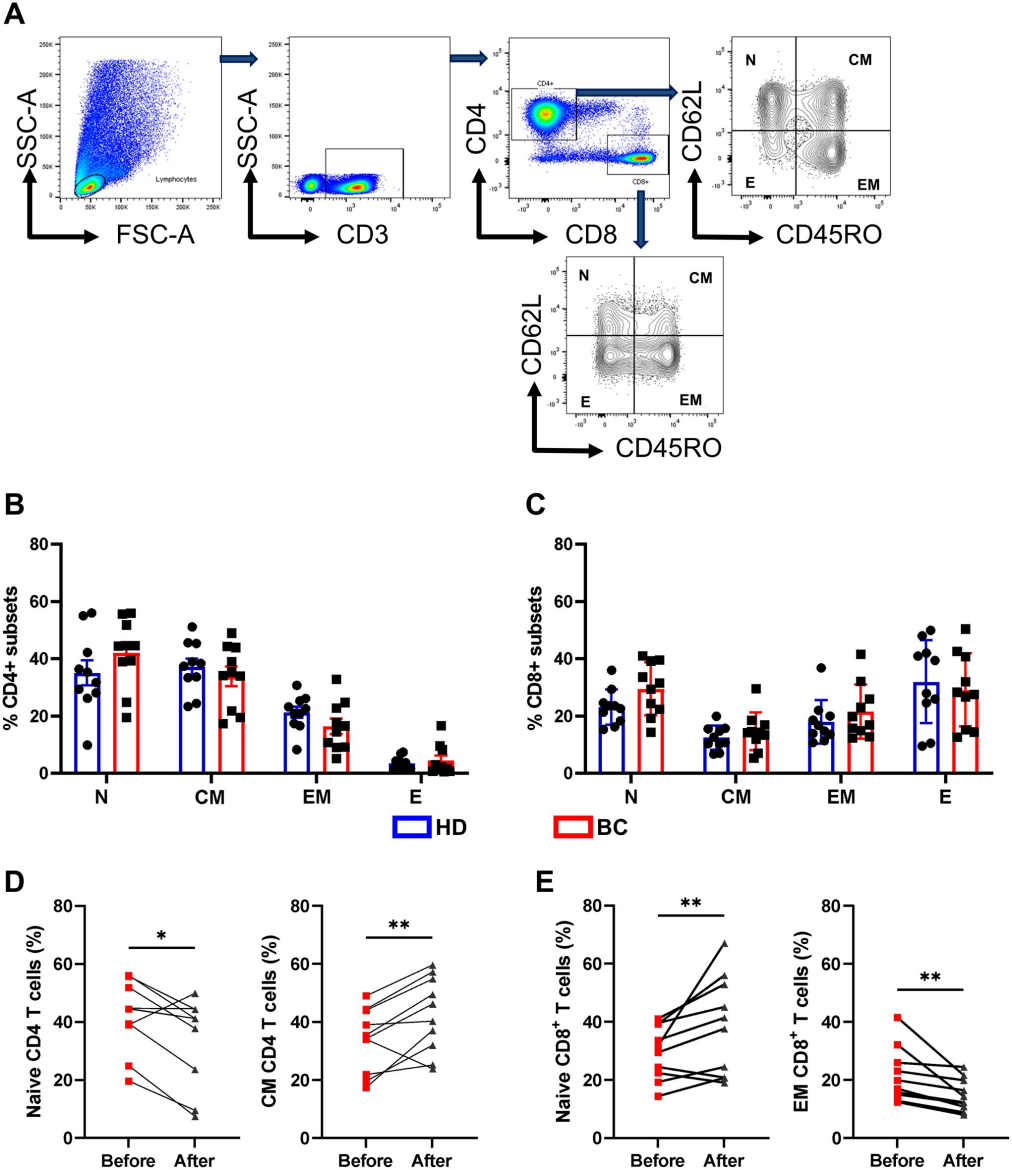
Change in CD4+ and CD8+ T cell subsets in chemotherapy-treated patients. **A**. Gating strategy used to identify memory population in T cells: CD62L+CD45RO− naïve T cells (**N** cells), CD62L+CD45RO+ central memory T cells (**CM** cells), CD62L−CD45RO+ effector memory T cells (**EM** cells) and CD62L−CD45RO− terminal effector T cells (**E** cells) within CD3+CD4+ and CD3+CD8+ compartments. **B**. Scatter point bars showing the percentage of each memory subpopulation in HD (blue box) vs. BC patient’s (red box) in CD4+ and **C**. CD8 + T cells. **D**. Frequencies of naïve and central memory T cells within total CD4+ compartments in BC patients before and after chemotherapy. **E**. Frequencies of naïve and effector memory T cells within total CD8+ compartments in BC patients before and after chemotherapy (n = 10/group). A nonparametric *t*-test was performed with unpaired Mann-Whitney test data to compare HD and BC patient’s. A paired nonparametric Wilcoxon test to compare BC patients before and after chemotherapy. Data presented as means ± SEM (** p <0.01; * p <0.05).

Although CD8+ T cells are more vulnerable to stress due to aging and latent chronic infections, such as CMV [25], it appears that the CD4+ T cell compartment may be irreversibly affected after chemotherapy treatment [26]. In this context, we evaluated the distribution of memory populations in CD4+ and CD8+ compartments in BC patients before and after NAC. To assess memory subpopulations, we used the differential expression of CD62L and CD45RO membrane receptors to identify the following T cell subsets: (a) CD62L+CD45RO− naive T cells (N cells), (b) CD62L+CD45RO+ central memory T cells (CM cells), (c) CD62L−CD45RO+ effector memory T cells (EM cells), and (d) CD62L−CD45RO− terminal effector memory T cells (E cells; Fig 2A). We found no significant differences in the frequency of memory subpopulations of T cells (CD4+ and CD8+) between BC patients and HD (Fig 2B and 2C). However, when we compared patients before and after chemotherapy, we discovered a decrease in the naive population and an increase in the CM cell population in CD4+ T cells from BC patients after chemotherapy (Fig 2D). In contrast, in the case of CD8+ T cells, the analysis revealed an increase in the naive cell population and a decrease in the EM population in patients after chemotherapy (Fig 2E).

### Patients with BC exhibit a T cells exhaustion that is recovered after treatment with NAC

**Fig 3:**
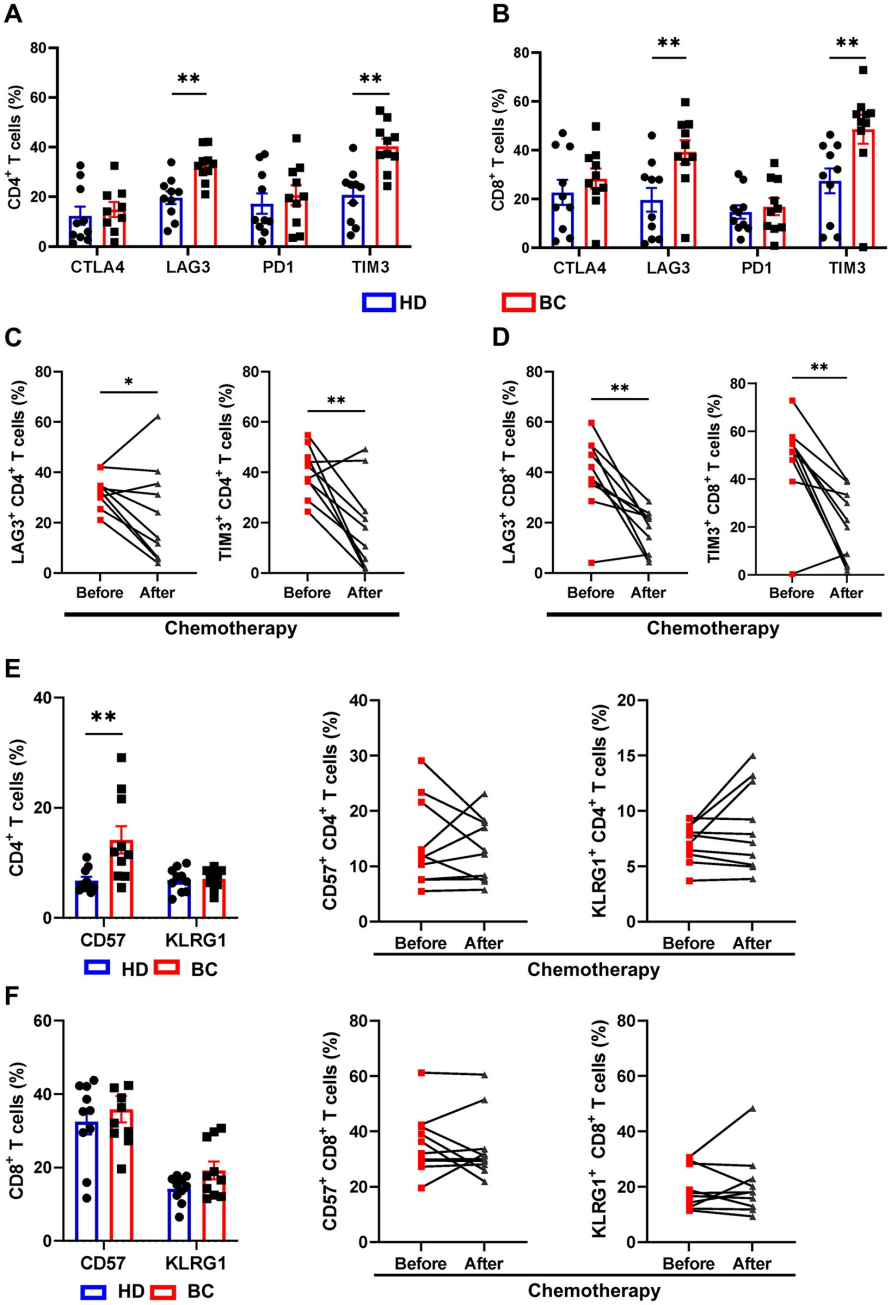
Change in senescence and exhaustion markers of T cells of BC patients. **A-B-**Exhaustion markers expression on CD4+ and CD8+ T cells, as assessed by flow cytometry on PBMCs from ten healthy donors (blue bars) and ten BC patients (red bars). **C-D**. Frequencies of LAG3+ and TIM3+ cells within total CD4+ and CD8+ T cells compartments in BC patients before and after chemotherapy. **E-F**. Senescence markers expression on CD4+ and CD8+ T cells, as assessed by flow cytometry on PBMCs from ten healthy donors (blue bars) and ten BC patients (red bars). Frequencies of CD57+ and KLRG1+ cells within total CD4+ and CD8+ T cells compartments in BC patients before and after chemotherapy. Data presented as means ± SEM (** p <0.01; * p <0.05).

In the literature, various mechanisms have been reported about how the TME impacts T cell function and phenotype, inducing T cell anergy, exhaustion, or senescence phenotypes as tumor escape strategies [14]. Antineoplastic drugs, such as anthracyclines, taxanes, and some platinum derivatives, among others, have been shown to achieve long-term clinical remissions in cancer patients partially by stimulating innate and adaptive immune cells [22]. However, the effect of NAC on the phenotype of T cell exhaustion or senescence induced by the TME and whether this can be evaluated in the peripheral blood of treated patients is not fully understood. To assess these effects, we used a multicolor panel of antibodies to measure the level of expression of exhaustion (CTLA4, PD1, TIM3, and LAG3) and senescence (CD57 and KLRG1) markers on T cells from BC patients before and after NAC using multicolor flow cytometry. We found that BC patients had higher expression of TIM3 and LAG3 in CD4 and CD8 T cells when compared with HD patients (Fig 3A and 3B). However, after chemotherapy, the expression of these two markers in the T cell compartment was significantly reduced (Fig 3C and 3D). On the other hand, when we evaluate senescence markers, the patients had a higher expression of CD57 in CD4 than HD (Fig 3E). Interestingly, we found no statistically significant change in CD57 expression associated with treatment (Fig 3E and 3F). We performed a differential analysis on the expression of senescence/exhaustion markers between the memory subsets of CD8 and CD4 T cells. We found that it follows the general trend of T cells (Fig 3). The naive population CD4 primarily expressed TIM3, whereas the subpopulations CM and EM of CD8 T cells expressed the most exhaustion markers (S3 Fig).

We calculated the residual cancer burden (RCB), which is derived from primary tumor dimensions, tumor bed cellularity, and axillary node burden and represents a validated score for informing the response and clinical efficacy of neoadjuvant chemotherapy to evaluate if T cell senescence marker expression and observed therapeutic effects correlate with patients’ ages [27, 28]. We divided the patients into two groups based on their RCB: strong responders (patients with minimal/moderate residual disease) and weak responders (patients with extensive residual disease). The mean age of the weak responders (RCB III) was higher (65.2) than that of the strong responders (RCB I/II, 55.5; S4A Fig). The relationship between RCB and age suggests that older patients are less responsive to therapy (S4B Fig).

When we evaluated the relationship between the expression of senescence markers and age, we found that older patients had higher levels of CD57 (S4C Fig). Following patient classification based on RCB score, a trend of higher expression of senescence markers in the weak responder group to NAC was found; this is evident mainly in CD4 T cells (S4D Fig). Similarly, we found that the weak responder group expresses more exhaustion markers before and after chemotherapy, particularly TIM3, which is evidenced mainly in the CD4+ T cell population (S5A Fig). However, after NAC, most patients have a decrease in the expression of exhaustion markers (LAG3 and TIM3; Fig 3D).

### Automatized analysis confirms a recovery of T cell exhaustion after NAC

**Fig 4:**
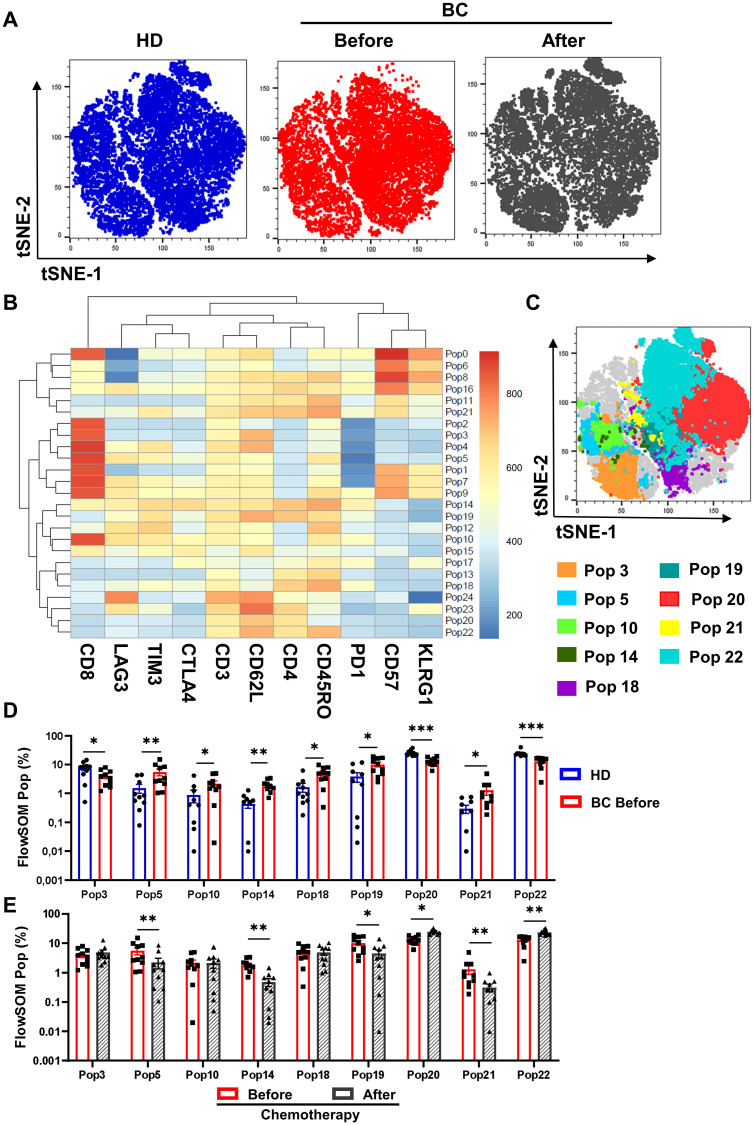
Identification of T-cell populations by FlowSOM. **A**. tSNE plots of concatenated samples of each group HD (blue), BC patients before chemotherapy (red), after chemotherapy (gray) **B**. Heat map of each marker in the 24 populations determined by FlowSOM. **C**. tSNE plots of concatenated samples with the relative location of the nine principal populations determined by FlowSOM that showed a difference among groups. **D**. Bar graph of relative cell frequency of nine populations determined by FlowSOM between HD (blue bars) and BC patients before chemotherapy (red bars). **E**. Bar graph of the relative cell frequency of nine populations determined by FlowSOM between BC patients before chemotherapy (red) and after chemotherapy (gray). A nonparametric *t*-test was performed with unpaired Mann-Whitney test data to compare HD and BC patient’s. A paired nonparametric Wilcoxon test to compare BC patients before and after chemotherapy. Data presented as means ± SEM (*** p <0.001; ** p <0.01; * p <0.05).

We used a dimensionality reduction algorithm with tSNE on BC patients and healthy donors to perform an unsupervised analysis. This analysis revealed distinct topological regions between HD and patients (before and after chemotherapy; Fig 4A). Subsequently, we used FlowSOM to perform cluster identification analysis, identifying 24 different T cell populations (Fig 4B). Nine of them revealed statistically significant differences between BC patients before NAC and HD (Fig 4C and 4D). Furthermore, when we compared BC patients before chemotherapy with HD, we found: (a) a decrease in the naive CD8+ T cell population (Pop3), (b) an increase in two central memory CD8+ T cell populations expressing exhaustion markers (Pop5 and Pop10), (c) an increase in two CM (Pop14 and Pop19) and one EM (Pop18) populations in the CD4 T cell compartment that expressed exhaustion markers, particularly TIM3, (d) an increase of CD4 T CM population with a senescent phenotype (Pop21), and (e) a decrease in naive and CM (Pop20 and Pop22, respectively; Fig 4D).

In terms of NAC’s effect in these populations, we observed an immunomodulatory effect on previously described Pop5, Pop14, Pop19, and Pop21 (Fig 4E), which decreased after treatment. Additionally, we noticed an increase in naive and CM populations (Pop20 and Pop22) that do not express exhaustion/senescence markers after NAC (Fig 4E).

**Fig 5:**
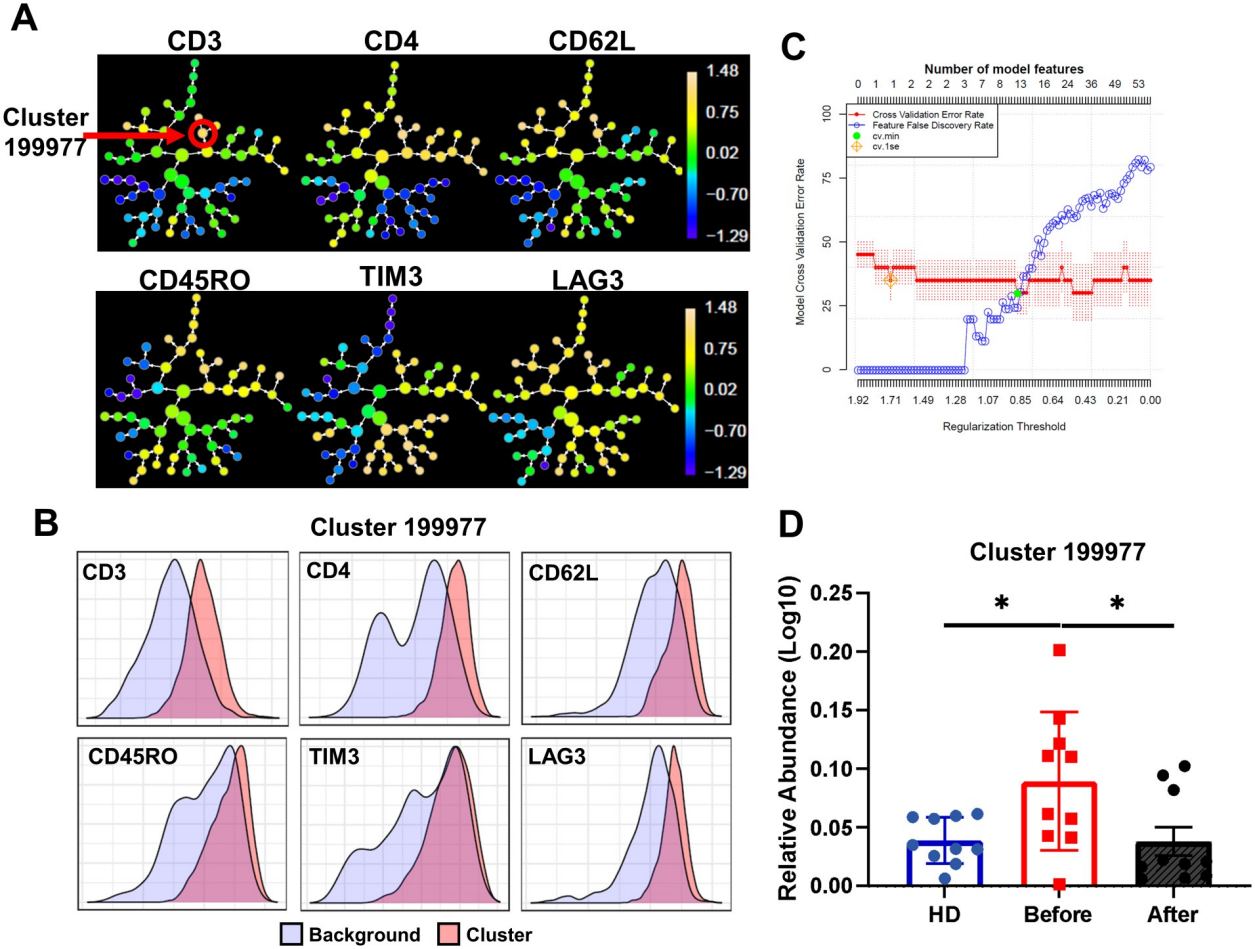
CITRUS analysis of T cell samples obtained from healthy donors (HDs), before, and after chemotherapy. **A**. Cluster distribution (hierarchy) and expression level of the corresponding marker from low (blue) to high expression (pink) for each marker, arrow points at cluster 199977. **B**. Histograms depicting the phenotype of cluster 199977 (red histograms) relative to background expression (blue histograms) for each marker (CD3, CD4, CD62L, CD45RO, TIM3, and LAG3. **C**. Cross validation error curves for number of models predicted by CITRUS, red curve: Cross Validation Error Rate, Blue Curve: Feature False Discovery Rate. Symbols represent best models CV. Min and CV 1SE. **D**. Relative abundance (Log10) of cluster 199977 in the three groups, HD (blue bars), patients before chemotherapy (red bars), and after chemotherapy (gray bars) (n = 10/group). A nonparametric *t*-test was performed with unpaired Mann-Whitney test data to compare HD and BC patient’s. Data presented as means ± SEM (* p <0.05).

To confirm these results, we performed an unsupervised analysis using CITRUS among HD, BC before, and after chemotherapy groups, which revealed a statistically significant increase in a cluster characterized by an exhaustion central memory CD4 phenotype in BC patients before chemotherapy (199977: CD3+ CD4+ CD62L+ CD45RO+ TIM3+ LAG3+; Fig 5A, 5B and 5D). A cross-validation test that specifies the feature false discovery rates (FDRs) was used to validate the number of models. The cross-validation error rate established that one model (cluster 199977) is significant (CV.1se) to differentiate the analyzed groups (HD, before and after chemotherapy) Fig 5C. This result confirms our previous results from manual (Fig 3A) and FlowSOM analysis (Fig 4B). Furthermore, we observed an increase in the cluster 59993: CD3+ CD4+ CD62L+ CD45RO− TIM3+ LAG3+ PD1+ CD57+ KLRG1+ before chemotherapy in a second cohort of patients (n = 3, S6 Fig).

## Discussion

Breast cancer patients exhibit various antitumor immune defects, including alterations in DC maturation, increased populations of Tregs and MDSCs, impaired cytotoxic functions of NKs, and T cell dysfunction [20]. Additionally, it has been reported that patients with BC exhibit a general suppression of the T cell and APC compartments, which NAC reverses, resulting in a better clinical response [22]. In this study, we use a similar model to monitor T cell responses to in vitro stimulation. We evaluated the TCR internalization as measured by loss of CD3 expression and found that T cells from BC patients have a significant decrease in response to in vitro stimulation, which is nearly restored after NAC at the same level as the control group [22].

According to several studies, the decrease in T cell response to TCR stimulation in terminally differentiated T cells is a consequence of alterations in the expression or phosphorylation of signaling pathway molecules, including CD3ζ and ZAP70 or results by activation of inhibitory receptors, such as CTLA4 [29–31]. Considering that T cells from BC patients exhibit a lower response to TCR stimulation, we analyzed ZAP70 phosphorylation in CD8+ T cells after in vitro stimulation. BC patients have lower ZAP70 phosphorylation, which is consistent with lower CD3 internalization in response to the stimulus. This T cell functional alteration has been described primarily in senescent CD8 T cells, which reduce their specific antigen response while increasing the expression of NK receptors [29]. This finding suggests that this alteration may reflect how the TME induces senescence in lymphocytes, affecting the circulating cells of BC patients. More research is needed to determine how senescence and exhaustion affect T cell activation capacity.

Senescence and exhaustion are characterized by a decrease in T cell proliferative capacity [16, 32]. Ki67 expression was measured in memory subpopulations of CD8+ T cells in this study. We found that terminal effector T cells from HD and BC patients have lower proliferative capacity than less differentiated subpopulations. It has been reported that immunosenescence and exhaustion signaling pathways play a significant role in T cell proliferative capacity. Henson et al. [32] demonstrated that suppressing the expression of PD1 (exhaustion) or p38 MAPK (senescence) in T cells could restore their proliferative capacity. Therefore, we can conclude that the different memory subpopulations of T cells in BC patients exhibit an exhausted phenotype with a significant decrease in their proliferation capacity. Exhaustion and senescence are dysfunctional states that differ from effector and memory states phenotypically and functionally [17]. When we use a multicolor panel of antibodies to measure the expression of exhaustion or senescence markers in T cells from BC patients, we found that after NAC, BC patients have a lower percentage of naive CD4+ T cells and a higher percentage of naive CD8+ T cells than healthy women. This finding is interesting because it has previously been reported that CD8 homeostasis is more vulnerable to stressors, such as aging and cytotoxic effect of chemotherapy [33, 34]. The increase in this naive subpopulation can be attributed to a different mechanism, including (a) thymic production, (b) homeostatic proliferation, and (c) naive T cell compartment repopulation due to infiltration of memory cells that tend to reacquire the naive-like (stem-like memory cells) phenotypeGustafson et al. found that chemotherapy induces naive CD8+ T cell recovery to normal levels by increasing the expansion of stem-like memory cells (CD95+). In contrast, the naive CD4+ subset has limited regeneration potential [26]; however, other studies have reported that naive T cells have a higher capacity to recover after chemotherapy than memory T cells [35].

Although exhausted and senescent T cells have similar dysfunctional features in antitumor immunity, their behavior differs during tumor progression in terms of development and metabolic regulation [17]. When we evaluated the expression of exhaustion markers (CTLA4, LAG3, PD1, and TIM3) in BC patients, we found that CD4+ and CD8+ T cells in exhibit a significant increase in TIM3 and LAG3 expression compared with HD. However, these exhaustion markers significantly decrease after treatment. We confirmed the findings of the manual analysis with an unsupervised multidimensionality reduction analysis. According to our manual analysis and what has been reported in patients with chronic infections and different types of cancer, we found increased CD4 (Pop 18 and Pop19) and CD8 (Pop5 and Pop10) exhausted clusters in BC samples [14]. Prolonged exposure of T cells to their cognate antigen induces signals of permanent TCR activation, increasing the expression of inhibitory receptors [36]. Furthermore, in response to stimulation, exhausted cells lose their effector functions and proliferation capacity and undergo various metabolic and transcriptional changes [15, 17, 36, 37].

PD1 and CTLA4 are the most studied inhibitory receptors. PD1 inhibits TCR activation by inhibiting ZAP70 and Lck phosphorylation; it also inhibits the PI3K/AKT/mTOR pathway, which is required for metabolic reprogramming, and reduces the proliferation and production of effector cytokines, such as IL-2, IFN, and TNF [17, 38]. CTLA4 binds to costimulatory molecules, such as CD80/86, and activates PP2A phosphatase, inhibiting Akt/mTOR signaling and causing T cells to enter the cell cycle [17]. Combining inhibitory receptor blockage, such as anti-PD1/PD-L1 and anti-CTLA4, has been shown to reverse LT depletion and improve immune responses in various types of cancer [39]. Unlike these two receptors, the intracellular signaling pathways of TIM3 and LAG3 have not been fully elucidated. TIM3 has the ability to interact with multiple components of the intracellular signaling complex of the TCR, affecting cellular functions [37]. LAG3 binds to CD3 and inhibits proliferation, cytokine production, and calcium flux [37]. Some studies have found correlations between poor clinical outcomes from antitumor treatment and the presence of exhausted tumor-infiltrating lymphocytes [40]. While there are no clear reports on the prognostic value of circulating exhausted T cells in BC patients, finding an immune signature in peripheral blood that can be measured by flow cytometry could help in the selection of a specific type of treatment [40].

As previously stated, an increase in the frequency of exhausted T cells in the TME has been associated with a poor prognosis in various types of cancer, emphasizing the importance of immunotherapy interventions that restore lymphocyte function [41]. In this study, we propose NAC treatment as a supplement to immunotherapy because we observed a decrease in the frequency of exhausted circulating T cells comparable with HD in patients who received it. Massa et al. [21] found a significant difference in PD1 expression in responders versus non-responders after two cycles of neoadjuvant chemotherapy in triple-negative BC patients, which is consistent with our findings. However, another study with patients with chronic lymphocytic leukemia found that chemotherapy increases the frequency of exhausted T cells, suggesting that this treatment approach may impair immunosurveillance in this type of tumor [42]. When evaluating the in-situ expression of such inhibitory receptors in BC tumors, some authors report significant decreases after NAC. For example, Kaewkangsadan et al. [43] reported a significant decrease in CTLA-4 in stromal T cells, as well as a decrease in intratumoral and stromal PD1+ T cells. Wang et al. [44] evaluated LAG3 expression in CD8+ T cells and showed an increase in its expression but in patients who did not respond favorably to NAC. Furthemore, Liang et al., [45] showed that TIM-3 tumors decreased only in non-major pathological response tumors. Others, such as Wesolowski et al., have characterized the immunogenic characteristics of tumor cells by evaluating their expression of these receptors, similar to Liang et al. [46] who found a decrease in the number of patients with tumors positive for total PD-1 and PD-L1 (both tumoral and stromal), Sarradin et al. [47] reported a significant decrease in stromal LAG3 expression after NAC treatment. Interestingly, Wu et al. revealed that chemotherapy induces an increase in T cell activation genes (CD28, CD27, CD86, and LCK levels) in older patients with BC, after 12 months of treatment, which was stronger in well-nourished patients and not observed in patients treated only with hormone therapy [48]. These studies and others [49–51] support our findings, indicating that chemotherapy restores T cell function, and that the subsequent immune system activation is related to a better clinical response to NAC.

The characteristics of the TME spatial/temporal investigation may explain the heterogeneity in findings supporting NAC’s immunostimulant effect. For example, Graeser et al. showed a decrease in tumoral PD-L1 expression after a single NAC cycle [52], which represents an early stage of therapy and could be related to tumor cell elimination. Moreover, they discovered an increase in PD1 expression in CD8+ T cells but not in CD4+ T cells; however, such an increase in PD1 expression in immune cells could also indicate T cell activation [53, 54]. On the other hand, chemotherapy may have different effects on intratumoral and peripheral T cell numbers and activation states. Cubas et al. found that most chemotherapeutic agents had an antagonistic effect on CD8+ T cells in the periphery, namely, the dLNs and blood, in a murine MC38 model, but this finding could be transient and did not translate into a reduction of the local antitumoral effect. Indeed, they discovered that cyclophosphamide increased the number of infiltrating CD8+ T cells and their activation state (IFNγ, ICOS, and GZMB) [55]. Accordingly, some clinical trials support the idea of immune induction by chemotherapy by demonstrating the synergistic effect of immunotherapy and chemotherapy [56–58].

T cell senescence has been proposed as a tumor-evasion mechanism [17, 59]. These cells exhibit the following characteristics: (a) decreased expression of costimulatory molecules, such as CD27 and CD28, (b) increased expression of CD57 and KLRG1, and (c) upregulation of senescence-associated β-galactosidase (SA-β-Gal) [17, 60, 61]. Unlike exhausted T cells, senescent cells are metabolically active. They can secrete various cytokines, such as IL-2, IL-6, IL-8, TNF, IFNγ, IL-10, and TGF-β [15, 32]. However, they cannot proliferate efficiently because they downregulate particular cell signaling molecules, such as Lck and LAT, while increasing the expression of NKRs and cell cycle arrest molecules, such as p16 and p21 [29, 62–64]. In this study, we found a higher expression of CD57 in the CD4+ T cells from BC patients and a population with a senescent phenotype in the central memory compartment of the CD4+ T cells of BC patients using unsupervised analysis.

Recent studies have reported that tumor cells can induce DNA damage responses in T cells, resulting in cell cycle arrest and cellular senescence [64]. Some even propose a different causal relationship in which premature aging can induce the development of BC. For example, Trintinaglia et al. showed that women with a history of childhood maltreatment had an increased senescent T cell population, which could be inducing cancer development or be less efficient in patrolling tumor development [65]. Regardless of the mechanism and cause of senescence, an increase in the senescent T cell pool is critical for decreased immunosurveillance, tumor development, and progression [64, 66]. The ability of tumor cells to induce exhaustion and senescence in T cells promotes tumor escape and progression. Studies have shown that the PD1-recruited SHP2 phosphatase prefers to dephosphorylate the co-stimulatory receptor CD28, suggesting that CD28 expression is required for an effective anti-PD1 treatment response in cancer [67, 68]. The accumulation of senescent CD28neg T cells in cancer patients may be detrimental to anti-checkpoint therapy. A better understanding of the mechanisms responsible for the induction of senescence and exhaustion in cancer patients is required for developing personalized cancer treatment and control strategies.

Finally, other studies have reported that NAC can accelerate aging and activate cellular senescence mechanisms [69]. Consistent with the findings of Onyema et al. [70], we observed an increase in the expression of CD57 CD4+ and KLRG1 in CD8+ T cells after treatment, primarily in terminally differentiated memory subpopulations (effector T cells). Therefore, it is possible that the cytotoxic effect of chemotherapy on lymphocytes with high replicative capacity, such as naive and central memory, can induce differentiation and aging in this population [70].

Bruni et al. reported an increase in senescent γδ terminally differentiated cells (CD57+ CD28− CD16+) with functional alterations in patients with metastatic colorectal cancer after chemotherapy [71]. Similarly, Sanoff et al. found that after NAC, T cells from BC patients increased expression of cellular senescence markers, such as p16, and secreted senescence-associated cytokines, such as VEGFA and MCP1 [69]. Chemotherapy has not been related to telomere shortening (a hallmark of aging) in T cells. On the contrary, it appears that its effect on cell aging occurs via DNA damage induction [72]. The study of senescence and exhaustion in immune cells, such as T cells, induced by tumor progression is important in the development of new treatments, such as immunotherapy and personalized vaccines, which rely on their ability to activate the immune system.

## Supporting information

S1 Fig The proliferation of memory subsets in CD4 T cells. (TIF)

S2 Fig Cytokine production by CD8+ T cells subpopulations. (TIF)

S3 Fig Expression of senescence and exhaustion markers in memory subsets of T cells. (TIF)

S4 Fig Analysis of the RCB score and RCB class with age and the expression of senescence markers. (TIF)

S5 Fig Analysis of the RCB score and RCB class with the expression of TIM3. (TIF)

S6 Fig CITRUS analysis of T cell samples obtained from the second cohort of breast cancer patients before and after chemotherapy. (TIF)
