# Supplementary figures and images for: Neoadjuvant chemotherapy modulates exhaustion of T cells in breast cancer patients
Source: PLoS One. 2023 Feb 10;18(2):e0280851. doi: 10.1371/journal.pone.0280851 (PMC9916600; doi:10.1371/journal.pone.0280851)

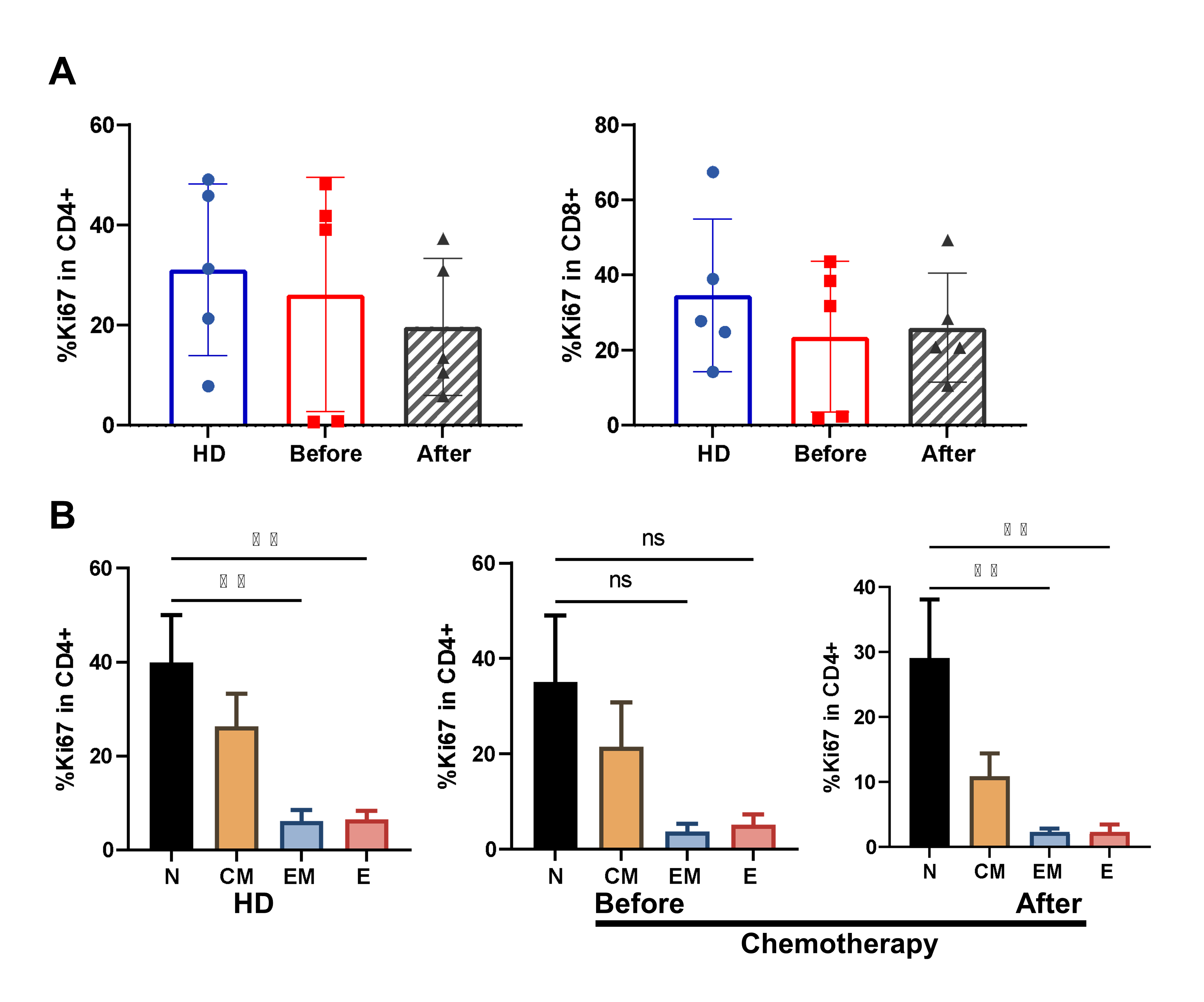

Supplement: S1 Fig — (TIF) [file pone.0280851.s001.tif]

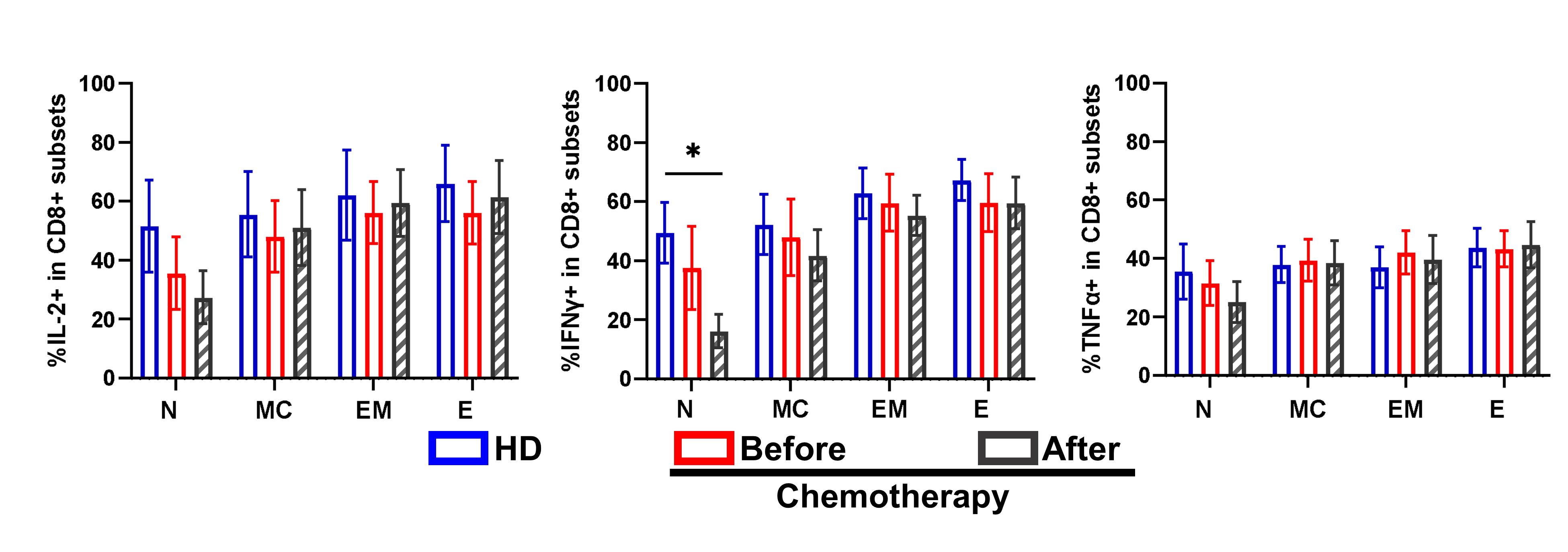

Supplement: S2 Fig — (TIF) [file pone.0280851.s002.tif]

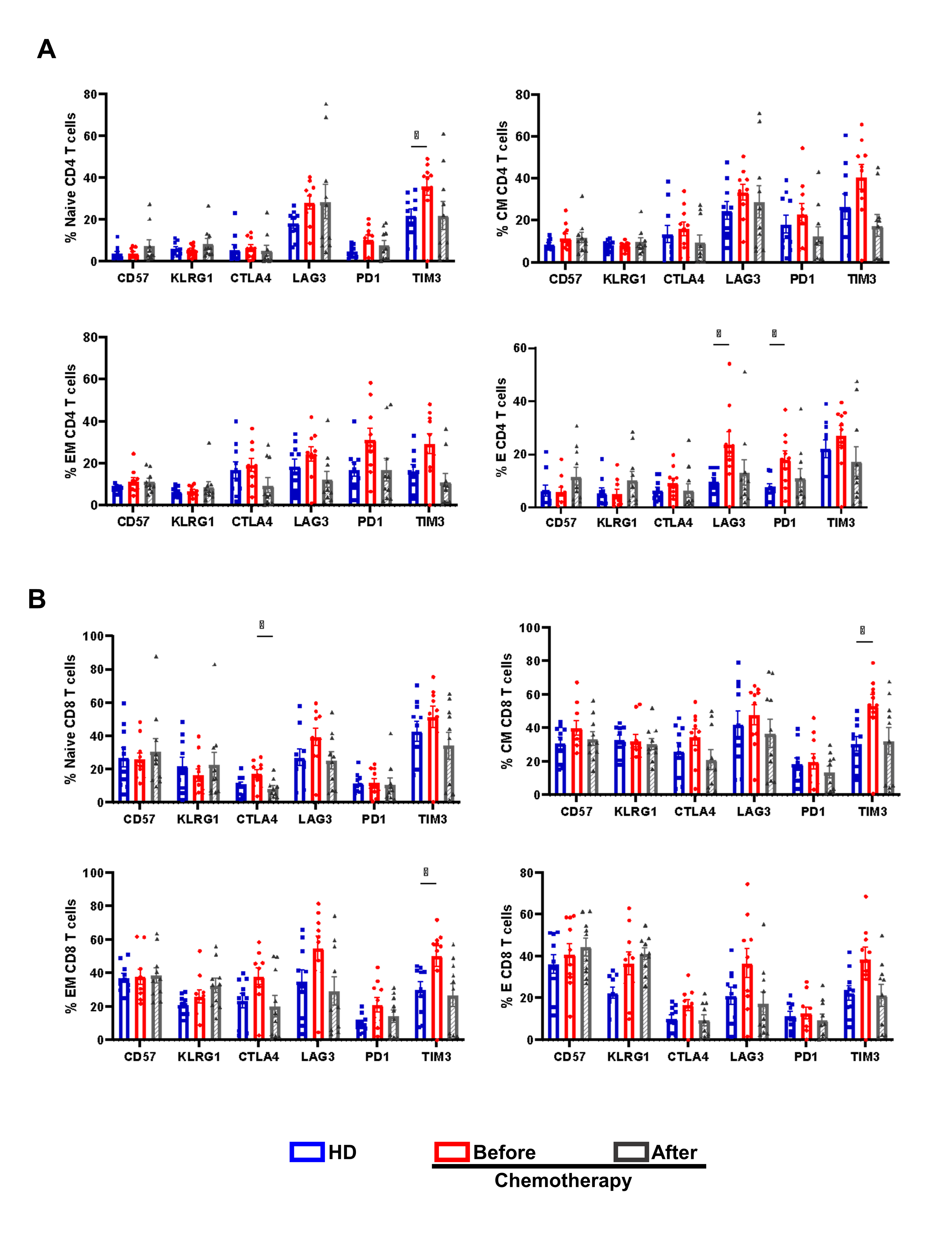

Supplement: S3 Fig — (TIF) [file pone.0280851.s003.tif]

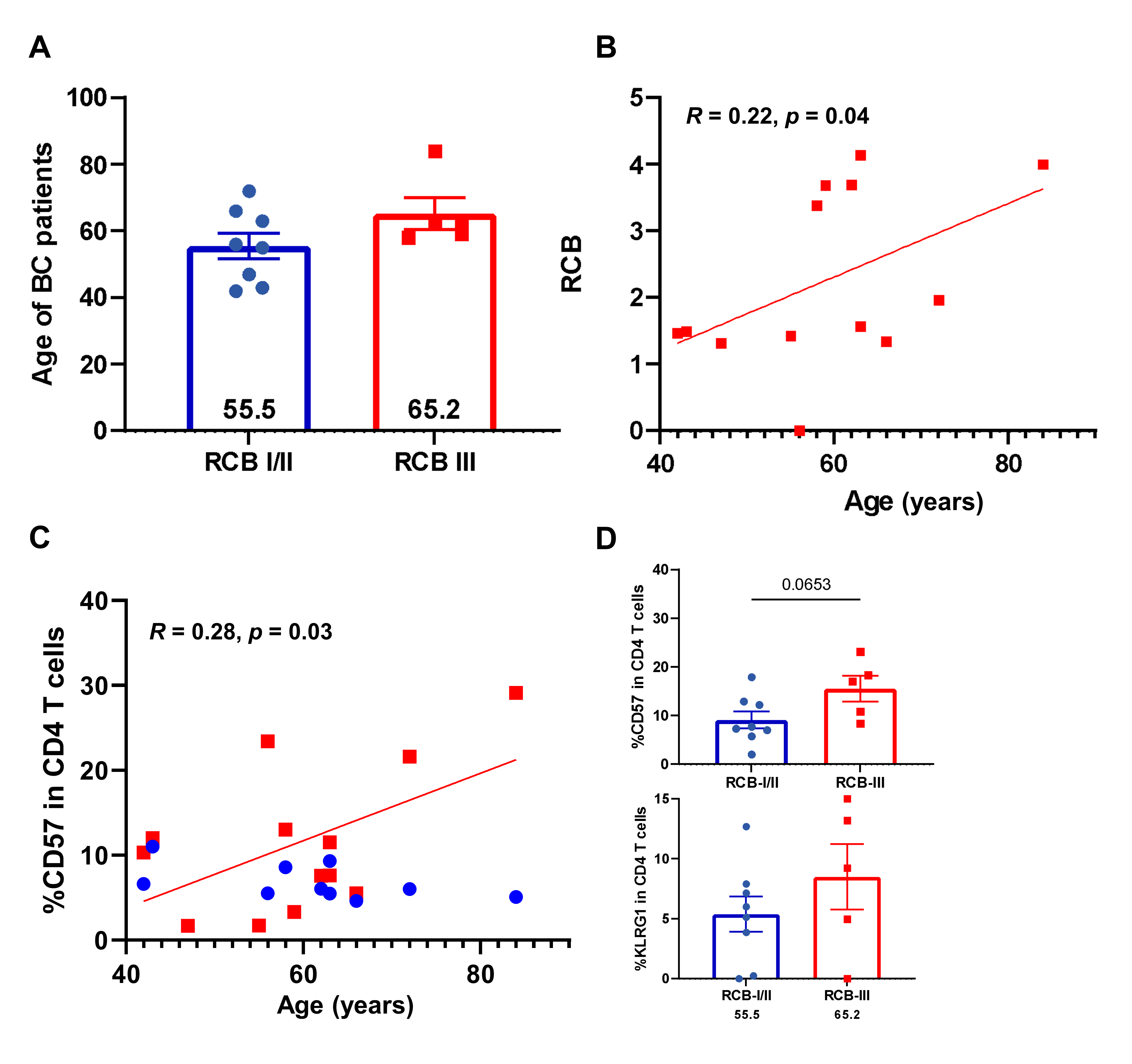

Supplement: S4 Fig — (TIF) [file pone.0280851.s004.tif]

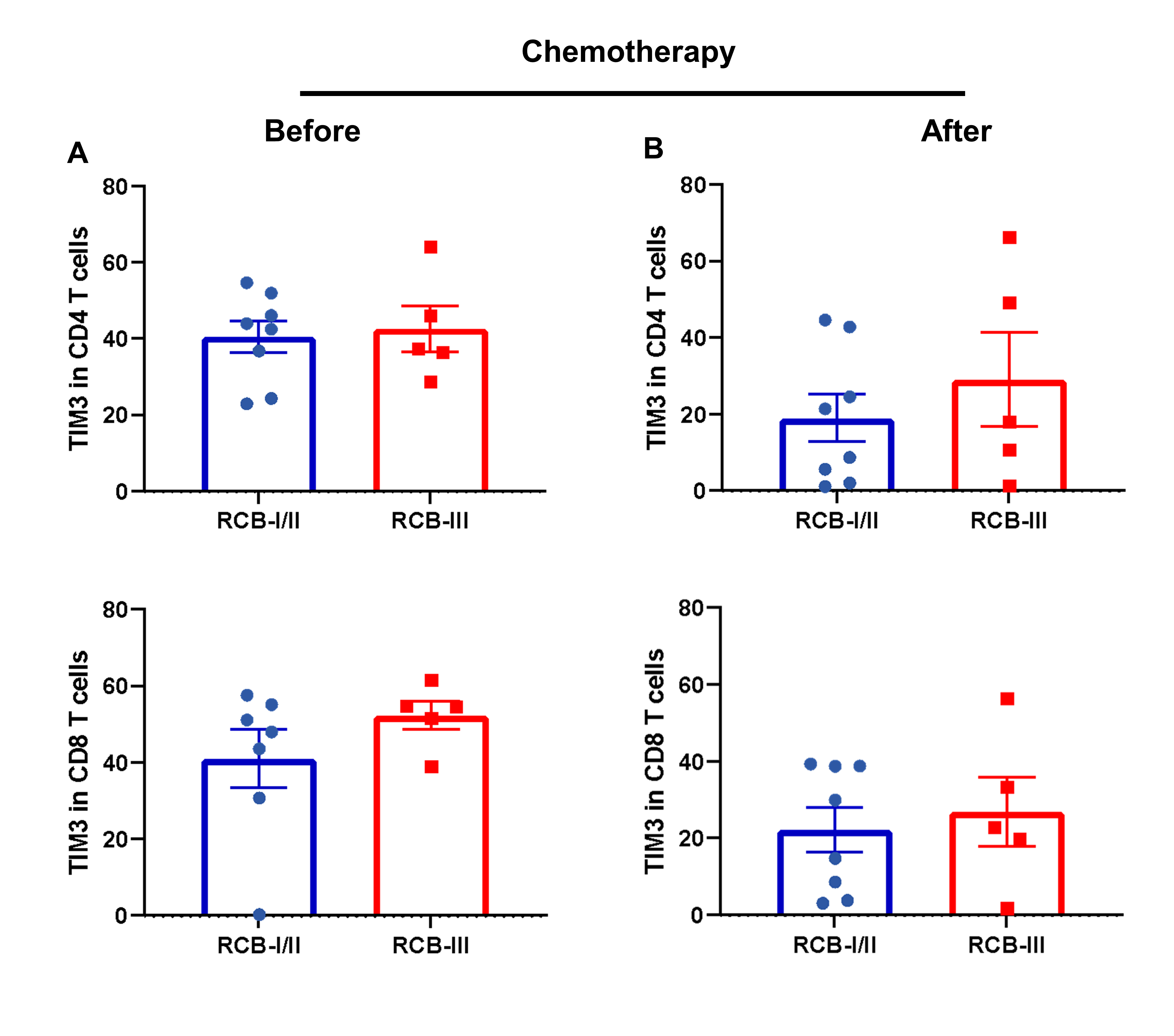

Supplement: S5 Fig — (TIF) [file pone.0280851.s005.tif]

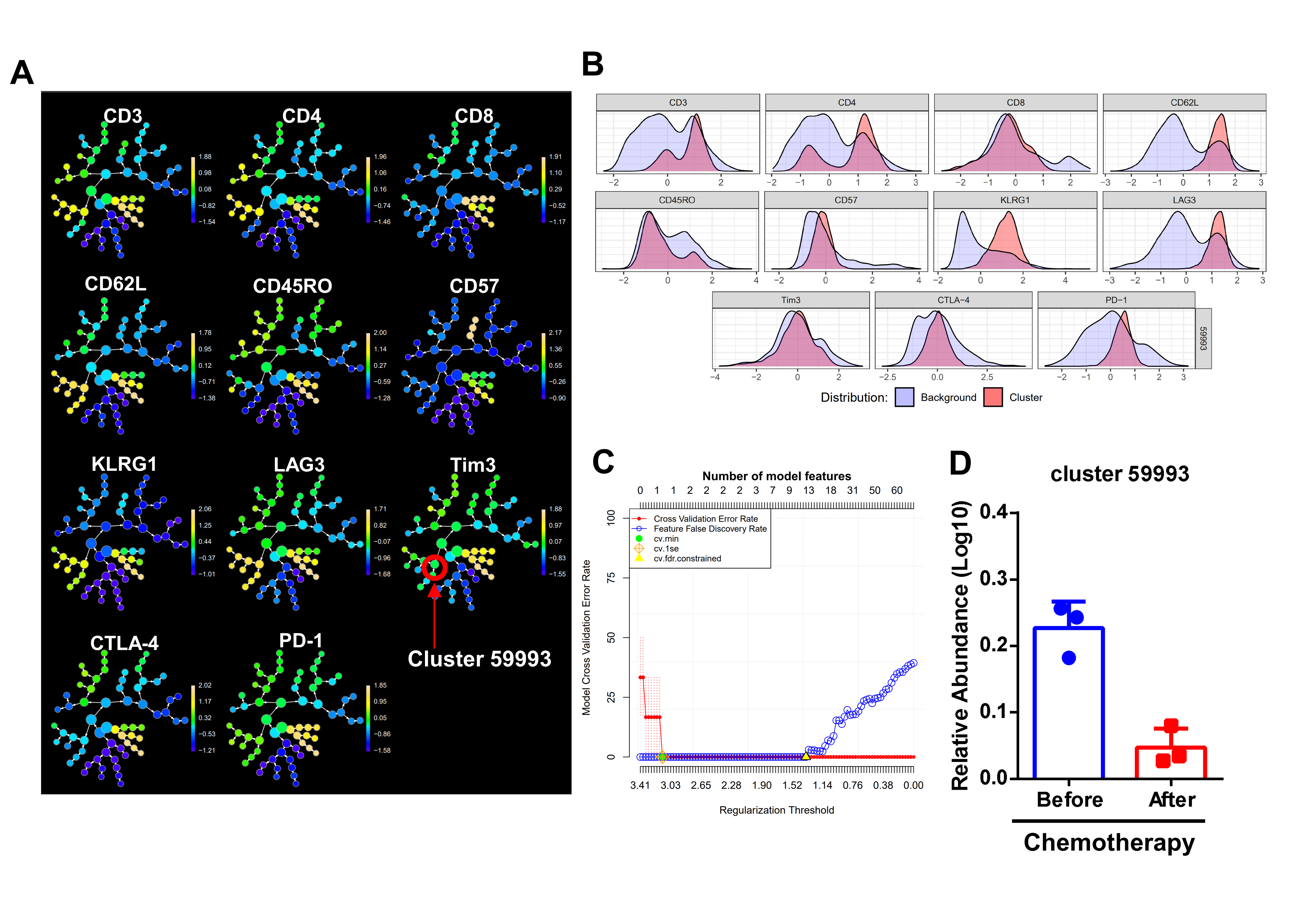

Supplement: S6 Fig — (TIF) [file pone.0280851.s006.tif]
